# Apparent Triclabendazole-Resistant Human *Fasciola hepatica* Infection, the Netherlands

**DOI:** 10.3201/eid1806.120302

**Published:** 2012-06

**Authors:** Annemarie J.S. Winkelhagen, Theo Mank, Peter J. de Vries, Robin Soetekouw

**Affiliations:** Kennemer Gasthuis, Haarlem, the Netherlands (A.J.S. Winkelhagen, R. Soetekouw);; Streeklaboratorium voor de Volksgezondheid, Haarlem (T. Mank);; Academic Medical Center, Amsterdam, the Netherlands (P.J. de Vries)

**Keywords:** Fasciola hepatica, fascioliasis, triclabendazole, drug resistance, the Netherlands, parasites, trematode

**To the Editor:** In December 2007, a 71-year-old sheep farmer sought care with a 4-month history of intermittent right upper quadrant pain, night sweats, anorexia, and a 5-kg weight loss. His medical history was unremarkable, and he had not traveled outside the Netherlands for ≈30 years. Physical examination revealed no abnormalities.

Blood tests showed an elevated erythrocyte sedimentation rate of 35 mm/h (reference 1–15 mm/h), normocytic anemia (hemoglobin 7.0 mmol/L [reference 8.5–11 mmol/L]), and eosinophilia (2.5 × 10^9^ cells/L [reference 0.0–0.5 × 10^9^ cells/L]). Levels of alkaline phosphatase, γ-glutamyl transferase, and alanine aminotransferase were elevated (146 U/L [reference 10–120 U/L], 143 U/L [reference 5–50 IU/L], and 54 U/L [reference 0–45 U/L], respectively). Levels of bilirubin and aspartate aminotransferase were normal. Computed tomography of the liver showed several irregularly shaped low-attenuating lesions ranging in size from 1 to 4 cm. High titers of IgG (640 [cutoff 40], determined by enzyme immunoassay) against *Fasciola hepatica* were detected. Subsequently, *F. hepatica* eggs were detected in fecal samples.

The patient, who spontaneously had become asymptomatic shortly after seeking care, was treated unsuccessfully with the benzimidazole derivative triclabendazole (TCBZ) on 3 separate occasions during the next 2 years. He was first treated with a single dose of 10 mg/kg TCBZ (Fasinex suspension; Novartis Animal Health Ltd., Surrey, UK), then with 2 doses 24 hours apart, and on the last occasion with 2 doses of TCBZ (Egaten; Sipharm Sisseln AG, Sisseln, Switzerland) 10 mg/kg 12 hours apart; the last 4 treatments were taken with food. Feces remained positive for *F. hepatica* eggs after each treatment. IgG titers remained positive (320, by enzyme immunoassay), and flukes could be visualized by ultrasound in the gallbladder and common bile duct (Figure). Thereafter, the patient was treated with nitazoxanide (500 mg 2×/d for 7 days); however, fecal samples remained positive for *F. hepatica* eggs. Lastly, after recent experiments of a combination therapy in a rat model (*1*), we treated the patient with TCBZ (Egaten, 10 mg/kg) combined with ketoconazol 10 mg/kg taken with food. Still, his feces remained positive for *F. hepatica* eggs.

**Figure:**
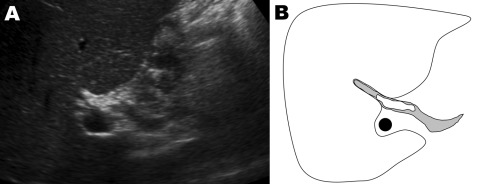
A) Ultrasound of the liver of a patient with *Fasciola hepatica* infection, the Netherlands. B) Drawing of A; depicted are the liver (white), the common bile duct (gray), and the portal vein (black). A fluke (white), measuring 2.5–3 cm long, is identified in the common bile duct.

Fascioliasis is a zoonotic disease caused by the foodborne trematode *F. hepatica* or *F. gigantica*, which has a complex life cycle and mainly affects sheep and cattle (*2*). Eggs of the adult worms (2–4 cm) that live in the bile ducts of the final host are excreted in the feces and develop into larvae (miracidia) in water. The miracidia then penetrate, and further develop in, snails of the family Lymnaeidae. Free-swimming cercariae exit the snail and attach to aquatic vegetation, where they encyst as metacercariae. After ingestion by the host, they excyst in the intestine and migrate through the intestinal wall to the liver, where they mature into adult flatworms that reside in the bile ducts (*2*).

Fascioliasis affects millions of humans worldwide (*3*); however, fascioliasis acquired in the Netherlands has been reported only sporadically (*4*), even though *F. hepatica* infection in sheep and cattle is prevalent there (*5*). The patient in this report had not eaten watercress or other aquatic plants and had not ingested ditchwater. However, he had worked in and around ditches on farms in the area, admitted chewing grass sporadically, and might have occasionally ingested vegetables previously fertilized with livestock manure. The patient remains asymptomatic but infected.

TCBZ is the treatment of choice for fascioliasis. In a review by Keiser et al. (*6*), the efficacy of treatment with TCBZ was shown to yield egg clearance in 78%–100% of patients after 1 dose of 10 mg/kg and in 92%–100% after 2 doses of 10 mg/kg each 12 or 24 hours apart. In livestock, TCBZ resistance is being reported increasingly (*7*). Mass treatment of sheep and cattle with TCBZ (Fasinex) or in combination with other anthelmintic drugs is common in the Netherlands (L. Moll, pers. comm.), and the first cases of resistance were described in 1998 in sheep and cattle in the province of North Holland, the area of residence of the patient reported in this study (*8**,**9*). During 1998–2004, resistance to TCBZ, proven by fecal egg count reduction tests, was found on 14 farms in the same area (*5*).

The findings in this case are most likely explained by TCBZ resistance, although we note that repeated TCBZ courses are not 100% effective against fascioliasis (*6*). Re-infection can be excluded because fecal samples were tested for eggs 1–3 months after each treatment. This description of apparent TCBZ-resistant fascioliasis in a human highlights the human health implications of (massive) anthelmintic use in livestock.

Further studies on TCBZ resistance and on therapeutics for fascioliasis need to be conducted. In addition, the role of antimicrobial drugs in the treatment of livestock needs to be more rigorously evaluated.
